# Comparison of the diagnostic value of liquid biopsy in leptomeningeal metastases: A systematic review and meta-analysis

**DOI:** 10.3389/fonc.2022.1079796

**Published:** 2022-12-19

**Authors:** Hanbin Wang, Luxuan Wang, Chuan Fang, Chunhui Li, Lijian Zhang

**Affiliations:** ^1^ Clinical Medicine College, Affiliated Hospital of Hebei University, Hebei University, Baoding, Hebei, China; ^2^ Department of Neurosurgery, Affiliated Hospital of Hebei University, Hebei University, Baoding, Hebei, China; ^3^ Department of Neurological Function Examination, Affiliated Hospital of Hebei University, Hebei University, Baoding, Hebei, China; ^4^ Postdoctoral Research Station of Neurosurgery, Affiliated Hospital of Hebei University, Hebei University, Baoding, Hebei, China; ^5^ Key Laboratory of Precise Diagnosis and Treatment of Glioma in Hebei Province, Affiliated Hospital of Hebei University, Hebei University, Baoding, Hebei, China; ^6^ Department of Reproductive Medicine, Affiliated Hospital of Hebei University, Hebei University, Baoding, Hebei, China

**Keywords:** brain parenchymal metastases, liquid biopsy, leptomeningeal metastases, diagnosis, meta-analysis

## Abstract

**Background:**

Brain metastases (BM) include brain parenchymal (BPM) and leptomeningeal metastases (LM), which are associated with a poor prognosis and high mortality rate. Early and accurate diagnosis and timely, effective treatment are crucial for improving the overall survival of LM patients. Cerebrospinal fluid (CSF) biopsy technology has attracted widespread attention for its diagnostic value in diverse cancers, including LM. We summarized studies to compare the potential diagnostic value of CSF liquid biopsy techniques in BM patients with meta-analysis.

**Methods:**

The study protocol was prospectively registered in PROSPERO, registration number CRD42022373263. We obtained the literature on liquid biopsy for BM from 7 databases (PubMed, Embase, Cochrane Library, Web of Science, China National Knowledge Infrastructure, and Wanfang Data knowledge service platform). Then, a systematic review of those studies was performed according to PRISMA criteria.

**Results:**

Nine publications have been obtained, and we found CSF liquid biopsy techniques to be more suitable for diagnosing LM. We analyzed the sensitivity, specificity, and area under the curve (AUC) of CSF liquid biopsy. The overall sensitivity, specificity, and AUC of CSF liquid biopsy in the diagnosis of LM were 0.65 (95% CI: 0.48 - 0.79), 0.70 (95% CI: 0.50 - 0.86), and 0.69, respectively. Then, we compared the diagnostic advantages of CSF liquid biopsy techniques and CSF cytology in LM. The results show that CSF liquid biopsy is superior to CSF cytology in LM diagnosis.

**Conclusions:**

Our meta-analysis suggested that CSF liquid biopsy is more suitable for LM diagnosis and has higher accuracy than CSF cytology.

## Background

Brain metastasis (BM) is an intracranial tumor that results from malignant tumors elsewhere in the body that metastasize to the brain. In the central nervous system (CNS) tumors, as a neurological complication of systemic malignancies, the incidence of BM accounts for about 20% of CNS malignancies (1). The rapid growth of BM can increase intracranial pressure and damage nerves, seriously jeopardizing the health and prognosis of patients (2). Therefore, early identification and diagnosis of BM are of prime importance.

Current advanced neuroimaging techniques could help to identify BM but are insufficient to make a definitive diagnosis (3). Surgical removal of tumor tissue followed by histopathological analysis remains the gold standard for diagnosing BM. However, collecting tumor tissue from malignancies of the CNS for purely diagnostic purposes is complex and risky. Compared with traditional tissue biopsy, liquid biopsy has attracted much attention because of its advantages such as simple operation, non-invasive and dynamic observation (4). Liquid biopsy can detect circulating tumor cells (CTCs), circulating tumor DNA (ctDNA), circulating cell-free DNA (cfDNA), and free circulating nucleic acids (mRNA and non-coding RNA) in the blood or cerebrospinal fluid of patients to diagnose the disease (5). There is evidence that the genomic characteristics of tumor tissue are critical to cancer diagnosis and treatment (6).

BM include brain parenchymal metastases (BPM) and leptomeningeal metastases (LM). Liquid biopsy techniques are of great value in diagnosing BM. However, studies have shown that fluid biopsy of cerebrospinal fluid (CSF) appears more promising in LM due to anatomical differences between BPM and LM (7). Therefore, to better understand the diagnostic value of CSF liquid biopsy in BPM and LM is of importance for the pre-operative diagnosis and decision-making in clinical settings. Here, we conducted a meta-analysis to compare the diagnostic value of CSF fluid biopsy for BPM and LM. In addition, we also analyzed the potential diagnostic value of CSF fluid biopsy in patients with LM.

## Materials and methods

### Search strategy

We scoured in PubMed, Embase, Cochrane Library, Web of Science, China National Knowledge Infrastructure (CNKI), and Wanfang Data knowledge service platform for published studies on liquid biopsy and brain metastases as of July 2022. We searched PubMed using the following strategy: (“brain metastasis”[Title/Abstract] OR “brain metastases”[Title/Abstract] OR “metastatic brain tumour”[Title/Abstract]) AND (“ctDNA”[Title/Abstract] OR “cfDNA”[Title/Abstract] OR “circulating tumor dna”[Title/Abstract] OR “circulating free dna”[Title/Abstract]). We searched Embase using the following strategy: brain metastasis OR brain metastases OR metastatic brain tumour AND ctDNA OR cfDNA OR circulating tumor DNA OR circulating free DNA. We searched Web of science using the following strategy: (TS=(brain metastasis) OR AB=(brain metastases OR metastatic brain tumour)) AND (TS=(ctDNA) OR AB=(cfDNA OR circulating tumor DNA OR circulating free DNA)).

### Eligibility criteria and quality assessment

Inclusion criteria were as follows (1): studies related to BM and liquid biopsy techniques (2); Provides the diagnostic ability of liquid biopsy technology for BM; (3) All patients with BM have been diagnosed; (4) Data related to BM and liquid biopsy techniques, including true positive (TP), false positive (FP), false negative (FN), and true negative (TN), can be extracted or calculated from the article. The exclusion criteria are: (1) non-human experimental studies; (2) Studies unrelated to BM and liquid biopsy techniques; (3) duplicate articles or data, as well as studies with insufficient data; (4) Abstracts, letters, editors, expert opinions, reviews, case report types of articles.

We independently assessed the quality of the included studies using the 9-star Newcastle-Ottawa Scale (NOS). We scored the included studies based on three aspects of the NOS assessment (selection, comparability, and results). We also assessed the quality of diagnostic studies by applying the diagnostic accuracy study quality assessment-2 (QUADAS-2) criteria. The QUADAS-2 included 14 items (8). Each domain is assessed based on the risk of bias and applicability. Each item must be answered with “yes”, “no” or “unclear”. Answering “yes” means that the risk of bias is low while answering “no” or “unclear” means that there is a potential risk of bias.

### Data extraction

Two reviewers independently extracted all data from the included studies using standardized forms and double-checked the data. We discussed all the disputes and had them resolved by a third reviewer.

### Statistical analysis

We used Review Manager 5.3 (The Nordic Cochrane Centre, The Cochrane Collaboration, London, UK) and Meta-disc to perform statistical analysis of all data. The sensitivity, specificity, positive and negative likelihood ratios (PLRs and NLRs), diagnostic advantage ratios (DORs), and 95% confidence intervals (CI) of the data were analyzed by applying Meta-disc software. We used *I^2^* to assess statistical heterogeneity. We used *I^2^* to assess statistical heterogeneity, with *I^2^* > 50% indicating significant heterogeneity and *I^2^* < 50% indicating that heterogeneity was not significant. If *I^2^* > 50%, the random effects model is used for analysis and further performed sensitivity analysis. If *I^2^* < 50%, the fixed-effect model is used for analysis without a sensitivity analysis. P values<0.05 was considered statistically significant.

## Results

### Study selection and study characteristics

**Figure 1:**
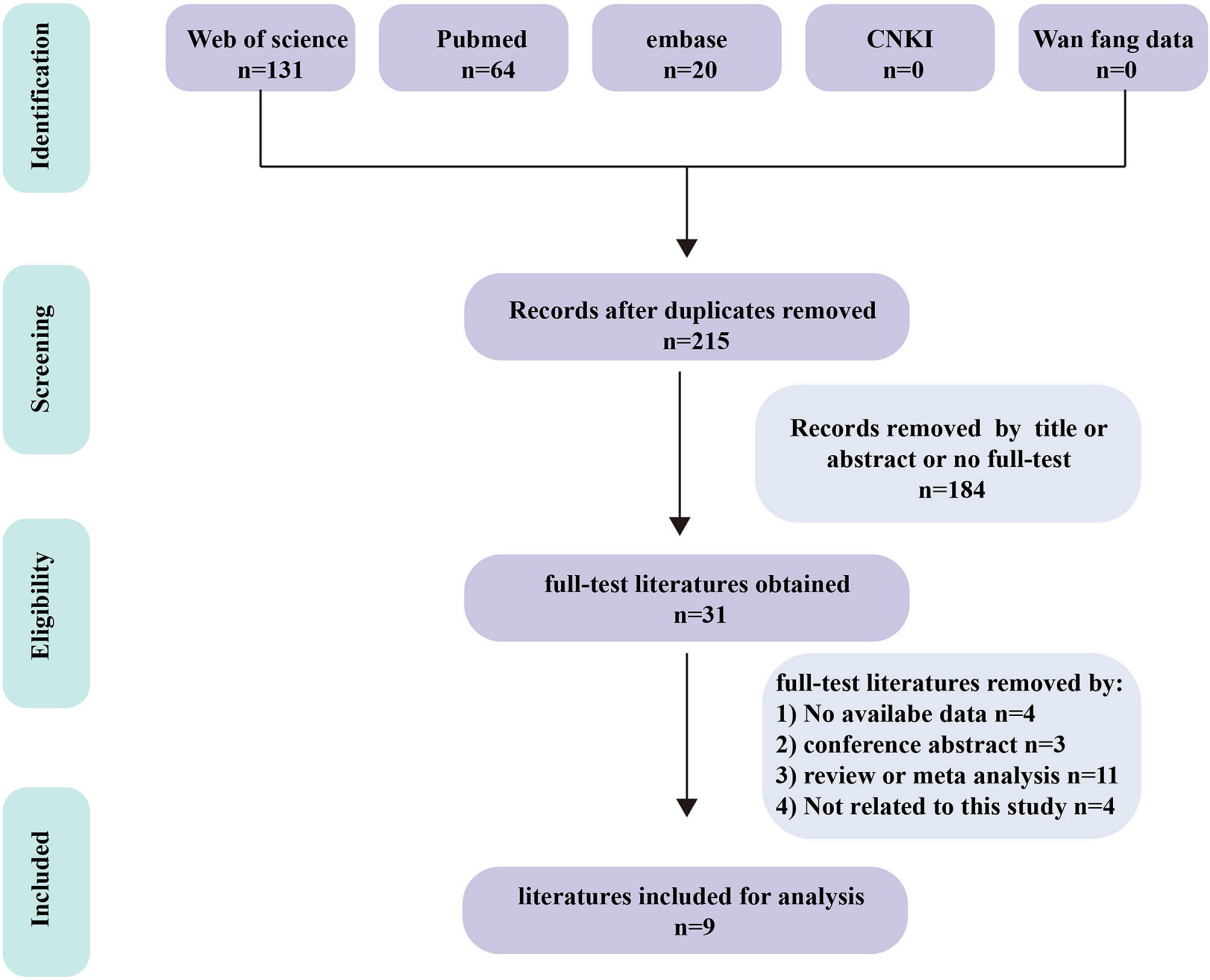
Flow diagram of the study selection for the present meta‐analysis.

We obtained 215 articles by searching databases such as PubMed, EMBASE, Web of Science, CNKI, and Wanfang Data knowledge service platform. By reviewing the title and abstract of the article, we excluded 3 duplicate articles and 179 articles that did not meet the inclusion criteria. We then read the full text of the remaining 33 articles, excluding 24 articles according to the exclusion criteria and incorporating 9 articles (9–17). The literature screening process for this study is shown in **Figure 1**. The nine articles are retrospective studies and come from different countries and regions. The nine articles included 191 patients with BM, including 53 patients with BPM and 138 with LM. In **Table 1**, we summarize the characteristics of the nine articles included in this study.

**Table 1:** Characteristics of studies included in this meta‐analysis.

Author, year	Source	Study design	Tumor Primary	Patients	Sample	Testing index (mutations)	Method	NOS	QUADAS-2
Cheok 2021	USA	Retrospective	NSCLC, SCLC	10	CSF cfDNA	EGFR, TP53, KRAS	error-suppressed deep sequencing	6	—
			Melanoma	2		TP53			
			Renal cell Carcinoma	1		NA			
			Colorectal cancer	1		KRAS			
Huang 2019	China	Retrospective	Lung Adenocarcinoma	22	CSF cfDNA	EGFR	ddPCR	6	11
Ma 2019	China	Retrospective	NSCLC	21	CSF ctDNA	EGFR	NGS	6	—
Pan 2015	USA	Retrospective	Colon Adenocarcinoma	1	CSF cfDNA	TP53	ddPCR	6	—
			Lung adenocarcinoma	3		EGFR			
			Melanoma	2		NRAS			
			NA	2					
Shah 2021	Texas	Retrospective	Breast cancer	17		TP53, CCND1, MYC, ERBB2/HER2	cytology	6	11
			Lung cancer	8		EGFR, TP53		0	
			Ovarian cancer	1		genetic alterations		0	
			Melanoma	1		genetic alterations		0	
			Uterine cancer	1		TP53		1	
			Breast cancer	17	CSF ctDNA	TP53, CCND1, MYC, ERBB2/HER2	Targeted NGS Assay	11	11
			Lung cancer	8		EGFR, TP53		3	
			Ovarian cancer	1		genetic alterations		0	
			Melanoma	1		genetic alterations		0	
			Uterine cancer	1		TP53		1	
White 2021	Boston	Retrospective	—	48			cytology	36	
				48	CSF cfDNA	cancer fraction	QIASymphony DSP Circulating DNA Kit	45	—
Ballester 2018	USA	Retrospective	Melanoma	7			cytology	4	
				7	CSF ctDNA	BRAF, NRAS, PIK3CA, ABL1, MET	ddPCR	6	11
Fitzpatrick 2022	UK	Retrospective	Breast cancer	24			cytology	12	
					CSF ctDNA	PIK3CA, E542K, TP53, MAF	ulpWGS	24	—
Momtaz 2016	USA	Retrospective	Melanoma	11			cytology	2	
					CSF cfDNA	BRAFV600E	ddPCR	6	—

NSCLC, Non-Small Cell Lung Cancer; SCLC, small-cell lung cancer; ddPCR, droplet digital polymerase chain reaction; NGS: Next-generation sequencing; ulpWGS, ultra-low pass whole genome sequencing; TAS, targeted amplicon sequencing; NA, not applicable.

### Quality assessment

The results of the quality assessment of all studies included in this meta-analysis are presented in **Table 1**. Studies that scored more than 6 points in NOS scores were considered of high quality. Studies that scored more than 11 points in the QUADAS-2 quality assessment were also considered high quality.

### Comparison of the diagnostic advantages of CSF liquid biopsy techniques in BPM and LM

**Figure 2:**
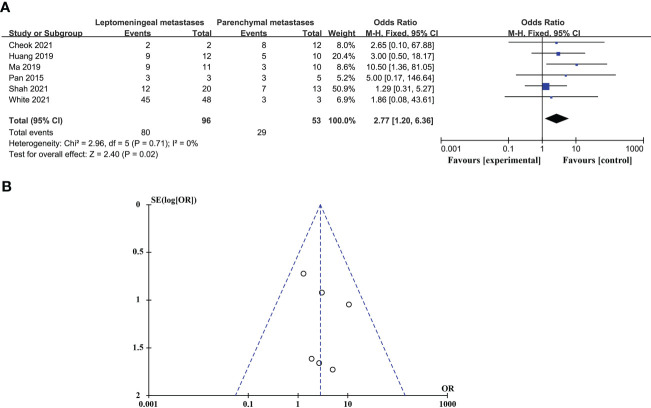
Comparison of the diagnostic advantages of CSF liquid biopsy techniques in BPM and LM. **(A)**. Forest plot of diagnostic advantages of CSF liquid biopsy techniques in BPM and LM. **(B)**. Funnel plot of diagnostic advantages of CSF liquid biopsy techniques in BPM and LM.

CSF biopsy techniques offer superior advantages in diagnosing LM than BPM, and the difference is statistically significant (OR_total_ = 2.77, 95%*CI*: 1.20 - 6.36). This analysis was performed using a fixed-effects model and did not require sensitivity analysis because there was no significant heterogeneity between studies (p = 0.71 > 0.05, *I^2^* = 0% < 50%). These data are shown in **Figure 2**.

### Pooled diagnostic value of CSF liquid biopsy for LM

**Figure 3:**
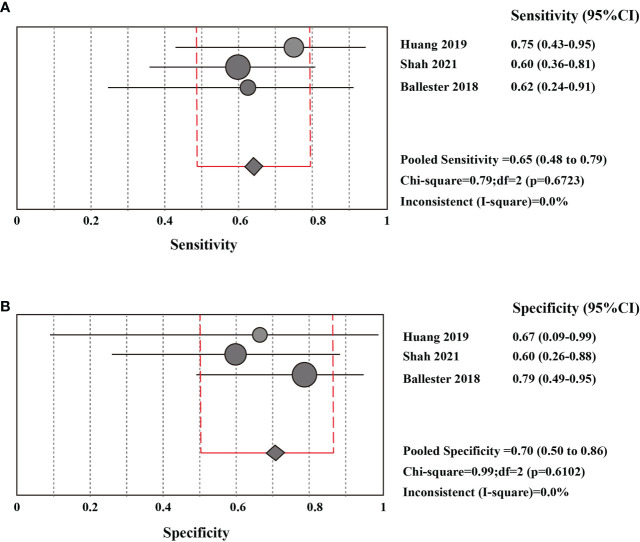
Pooled diagnostic value of CSF liquid biopsy for LM **(A)**. Pooled sensitivity of CSF liquid biopsy in diagnosis of LM. **(B)**. pooled specificity of CSF liquid biopsy in diagnosis of LM.

**Figure 4:**
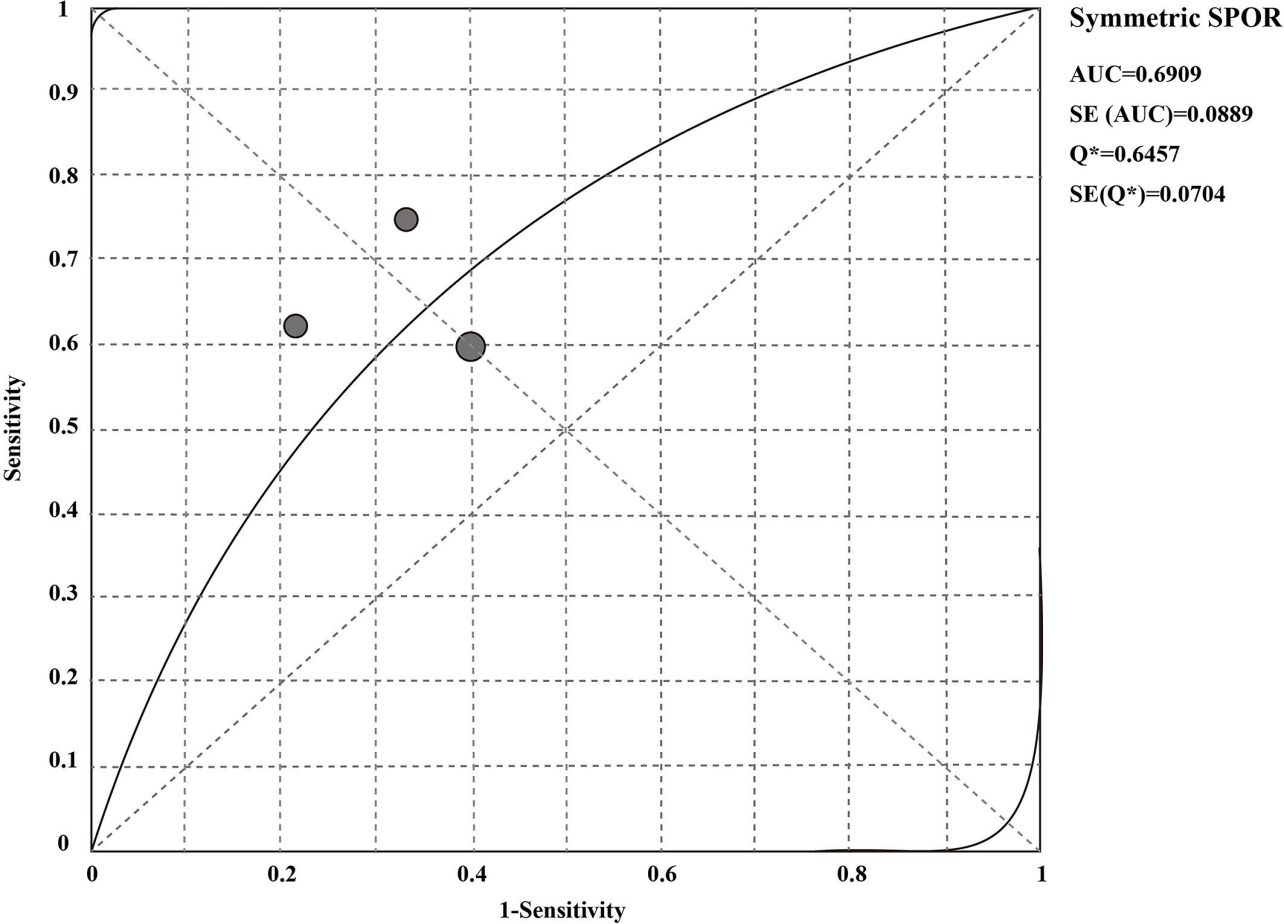
SROC curve of CSF liquid biopsy in the diagnosis of LM.

The sensitivity and specificity of CSF liquid biopsy for the diagnosis of LM are shown in **Figures 3A, B**. We use a fixed-effects model to pool sensitivity and specificity because *I^2^* < 50%. The sensitivity and specificity of the overall pooled were 0.65 (95% CI: 0.48 - 0.79) and 0.70 (95% CI: 0.50 - 0.86), respectively. The pooled PLR was 1.94 (95%CI:1.04 – 3.63), NLR was 0.54 (95%CI:0.32 - 0.92), and DOR was 3.67 (95%CI: 1.22 - 11.07) (**Supplementary Figures 1**–**3**). In addition, we also plotted an SROC curve to assess the accuracy of the diagnosis (**Figure 4**). The AUC is 0.69, indicating that the CSF liquid biopsy has a certain degree of diagnostic accuracy.

### Compare the diagnostic advantages of CSF liquid biopsy techniques and CSF cytology in LM

**Figure 5:**
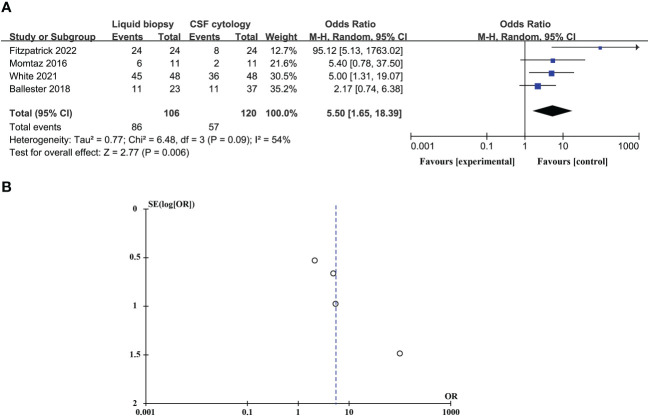
Compare the diagnostic advantages of CSF liquid biopsy techniques and CSF cytology in LM **(A)**. Forest plot of diagnostic advantages of CSF liquid biopsy techniques versus CSF cytology in LM. **(B)**. Funnel plot of diagnostic advantages of CSF liquid biopsy techniques versus CSF cytology in LM.

CSF liquid biopsy is superior to CSF cytology in the diagnosis of LM, and the difference is statistically significant (OR_total_ = 5.50, 95%CI: 1.65 – 18.39). Because of significant heterogeneity between studies (p = 0.09 > 0.05, *I^2^* = 54% > 50%), random effects models were used. These data are shown in **Figure 5A**. Evaluation potential publication bias using RevMan-generated funnel visualizations (**Figure 5B**).

### Sensitivity analysis and publication bias

**Figure 6:**
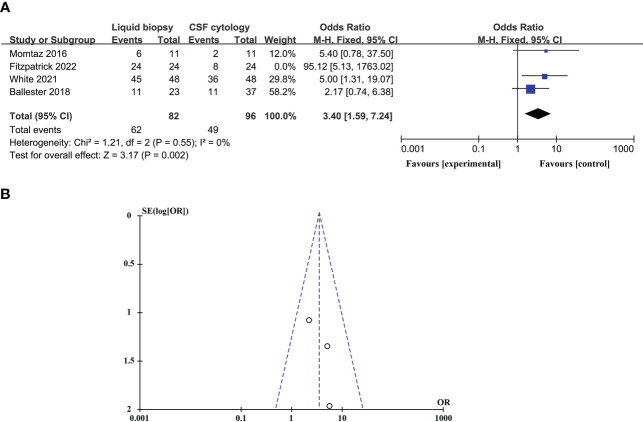
A Sensitivity analysis and publication bias. **(A)**. Forest plot of diagnostic advantages of CSF liquid biopsy techniques versus CSF cytology in LM after removal of heterogeneous cohorts by sensitivity analysis. **(B)**. Funnel plot of diagnostic advantages of CSF liquid biopsy techniques versus CSF cytology in LM after removal of heterogeneous cohorts by sensitivity analysis.

In studies comparing the diagnostic advantages of the CSF liquid biopsy technique and CSF cytology in LM, there was significant heterogeneity between studies (p = 0.09 > 0.05, *I^2^* = 54% > 50%), so we need to perform a sensitivity analysis. Sensitivity analysis found that Fitzpatrick’s research data was a dominating source of heterogeneity. After excluding Fitzpatrick’s studies, there was no statistical heterogeneity between the studies (p = 0.55 > 0.05, *I^2^* = 0% < 50%). This meta-analysis, again analyzed using fixed-effects models, showed that CSF liquid biopsy was still superior to CSF cytology in LM diagnosis (OR_total_ = 3.40, 95%CI: 1.59 – 7.24) (**Figure 6A**). Evaluation potential publication bias using RevMan-generated funnel visualizations. However, the number of included studies was too small, limiting the interpretability of the findings, as shown in **Figure 6B**.

## Discussion

As the incidence of BM continues to rise, people need an effective and convenient diagnostic method. Recently, researchers are paying attention to liquid biopsy technologies (18). The primary biological fluids associated with CNS malignancy studies include plasma and cerebrospinal fluid. CSF has many advantages over plasma, such as quiescent CSF is paucicellular and has low background levels of cell-free DNA (19). Studies have shown that CNS tumor-derived (ctDNA) is hard to detect in plasma, and the concentration of CNS tumor-derived (CTCs) in peripheral blood is much lower than that in CSF (20). The level of cfDNA related to intracranial diseases in plasma is low, and CSF circulating through the CNS seem superior to plasma in recognizing tumor-associated DNA (21). Though studies have emphasized the predictive power of CSF-based liquid biopsy with BM, there is still a pressing need to determine the diagnostic role of different liquid biopsy techniques in BM.

Metastases involving the CNS could be categorized into BPM and LM. In this meta-analysis, we compared the diagnostic power of CSF liquid biopsy between BPM and LM. The results showed that CSF liquid biopsy technology has advantages over BPM in diagnosing LM, which may be related to the anatomical structure of CNS. The CNS encompasses two distinct anatomic compartments: the densely cellular parenchyma and the CSF-filled leptomeningeal space (7). Unlike BPM, LM is present in the anatomical chamber containing cerebrospinal fluid. CSF sampling directly samples relevant space. Cells that metastasize to the leptomeningeal can be present in the CSF space, and the composition of CSF in LM patients is more susceptible to significant changes in the influence of cancer cells (22, 23). Zheng et al., demonstrated that CSFs of LM patients are rich in genome information related to LM, which makes CSF an ideal medium for evaluating LM (24). Moreover, previous studies also support our results that CSF fluid biopsy may be more suitable for LM diagnosis. Evidences have demonstrated that lung cancer, melanoma, and breast cancer are the most common primary tumors that metastasize to the brain, and they are also the most common causes of LM (25–27). Lung cancer is responsible for approximately 50% of all brain metastases. Mutations in the epidermal growth factor receptor (EGFR) gene are among the most common mutations in lung cancer (28). Huang et al. employed liquid biopsy technology to detect EGFR mutations in the CSF of lung cancer patients with BPM or LM. Their results indicated that EGFR mutations in the cerebrospinal fluid of LM patients were more detectable than those with BPM (13). Li and his colleagues also emphasized that CSF cfDNA could reveal the unique genetic profiles of LM and should be considered as the most representative liquid biopsy medium for LM in EGFR-mutant NSCLC (29). Breast cancer (BC) is the second most common solid tumor that can metastasize to CNS. Approximately 30% of patients develop BPM (30), and other 5% LM (31). Previous studies have showed the utility of CSF liquid biopsy to detect the clinically relevant genomic alterations in both LM and BPM (32, 33). However, there is still no evidence showing the priority of CSF liquid biopsy between BC patients with LM and BPM. For patients with melanoma LM, studies have shown that CSF liquid biopsy has a fine diagnostic and evaluation ability for LM (14). In addition, in a case report of patient with simultaneous occurrence of LM from both a pulmonary adenocarcinoma and malignant melanoma, Stoppek et al. confirmed the feasibility and diagnostic value of CSF liquid biopsy (34). Malignant tumors of the digestive tract, uterus, and ovary could also rarely metastasize to the brain (35). And they are the rarer forms of brain metastases. Thus, the literatures reporting their diagnostic powers are limited. Herein, our result suggested that CSF liquid biopsy might be more effective in diagnosing LM than BPM. However, there is no study directly compared the diagnosis power of CSF liquid biopsy among LP originated from different primary cancer sites which require investigation in the future study.

Further, we analyzed the potential diagnostic value of CSF liquid biopsy for LM. We found an overall combined sensitivity of 0.65 (95% CI: 0.48 - 0.79), specificity of 0.70 (95% CI: 0.50 - 0.86), and AUC of 0.69 for the CSF liquid biopsy technique in the diagnosis of LM. The results suggest that CSF biopsy has a certain degree of sensitivity, specificity, and diagnostic accuracy in LM diagnosis. DOR is the value of the combination of sensitivity and specificity, and the higher the DOR value, the better the diagnostic ability. The DOR is 3.67 (95%CI: 1.22 - 11.07), indicating that the overall merger accuracy is not high. The pooled PLR was 1.94, indicating a 1.94-fold increase in the probability of LM occurring when CSF liquid biopsy was positive. In addition, the NLR was 0.54, which means that when the CSF liquid biopsy was negative, the probability of developing LM increased by 46%. PLR>10 and NLR< 0.1 represents a high diagnostic accuracy (36). These results indicate that the diagnostic accuracy of CSF liquid biopsy may not be as high as expected. However, this does not mean that the CSF liquid biopsy technique is not valuable in LM diagnosis. The diagnosis of LM is complex, and LM can be identified by detecting the presence of malignant cells in the CSF or, in the absence of malignant cells in the CSF, by the concomitant characteristic clinical symptoms or signs and typical MRI findings (37–39). Clinically, the gold standard for LM diagnosis is CSF cytology, but even if CSF cytology is the gold standard, only 50% of cases show positive CSF cytology (40, 41). Therefore, to further clarify the diagnostic value of CSF liquid biopsy for LM, we compared the diagnostic advantages of cerebrospinal fluid biopsy and cerebrospinal fluid cytology in LM. The results indicate that CSF liquid biopsy is superior to CSF cytology in the diagnosis of LM (OR_total_ = 5.50, 95%CI: 1.65 – 18.39). We supposed that that CSF liquid biopsy is more accurate and valuable in diagnosing LM. Some previous studies also support our results. De Mattos-Arruda et al. found that CSF ctDNA analysis is more sensitive than CSF cytology in detecting LM (21). White et al. found that CSF cfDNA analysis may be more sensitive than CSF cytology in detecting LM (10). CSF liquid biopsy has great potential to facilitate and supplement the diagnosis of LM because of the low sensitivity of CSF cytology, particularly in cases that cannot be detected by conventional cytopathological analysis. Compared with CSF cytology, CSF liquid biopsy has many other advantages. In addition to the diagnosis of LM, the CSF liquid biopsy can better reflect the heterogeneity of the tumor and allow real-time monitoring of the evolution of cancer (4). Among ALK-rear-racking NSCLC combined LM patients, CSF liquid biopsy can be more sensitive to targeted changes and monitor tumor reactions (24). Based on the observation of this study, the CSF liquid biopsy showed improved diagnostic accuracy and sensitivity compared with cytology, and could provide molecular and genetic analysis of tumor associated markers or gene mutations. As to the liquid biopsy, many studies employ ddPCR in the clinical settings which provide accurate and reliable CSF analysis (14). Targeted sequencing method could be used to identify oncogene and tumor suppressor mutations. However, they focused on a limited number of genes, and may not represent the full spectrum of clinically relevant oncogenic drivers (42). With the advance in technological platforms development, expanding the number of genes and improving the sensitivity to detect mutations could improve the sensitivity of liquid biopsies in patients with BM (5).

There are some limitations to our study. First, despite our efforts to search for relevant studies, we may still have overlooked some studies that were not published online. Second, studies with positive results are more likely to be published, which may improve the accuracy of the overall diagnosis. Third, because of the CSF liquid biopsy technique is a broad concept, there are different methods of detecting CSF among the included studies, which may increase heterogeneity among the included studies and affect the reliability of these findings. In addition, the primary tumors and test indicators of LM patients included in this study may be different, which may also cause heterogeneity between studies and affect the reliability of the results. Finally, the small number of studies included in this study leads to the limited statistical power of these results.

## Conclusion

Our results suggested that CSF liquid biopsy are more suitable for diagnosing LM than that in BPM. In addition, we found that the overall diagnostic value of CSF liquid biopsy is low, and the diagnostic accuracy for LM is not as high as expected. However, by comparing the diagnostic advantages of the CSF liquid biopsy and CSF cytology in LM, our result showed that CSF liquid biopsy is superior to CSF cytology. Future studies are warranted to confirm our analysis.

## Author contributions

Conception and design: CL, LZ. Acquisition of data: HW, LW. Drafting the article: HW, LW. Critically revising the article: CL, LZ, and CF. All authors contributed to the article and approved the submitted version.

## Supplementary material

Supplementary Figure 1 The positive likelihood ratio of CSF liquid biopsy for LM diagnosis

Supplementary Figure 2 The negative likelihood ratio of CSF liquid biopsy for diagnosis of LM.

Supplementary Figure 3 Diagnostic odds ratios for the diagnosis of LM by CSF liquid biopsy.
