# Supplementary figures and images for: Comparison of the diagnostic value of liquid biopsy in leptomeningeal metastases: A systematic review and meta-analysis
Source: Front Oncol. 2022 Dec 19;12:1079796. doi: 10.3389/fonc.2022.1079796 (PMC9806138; doi:10.3389/fonc.2022.1079796)

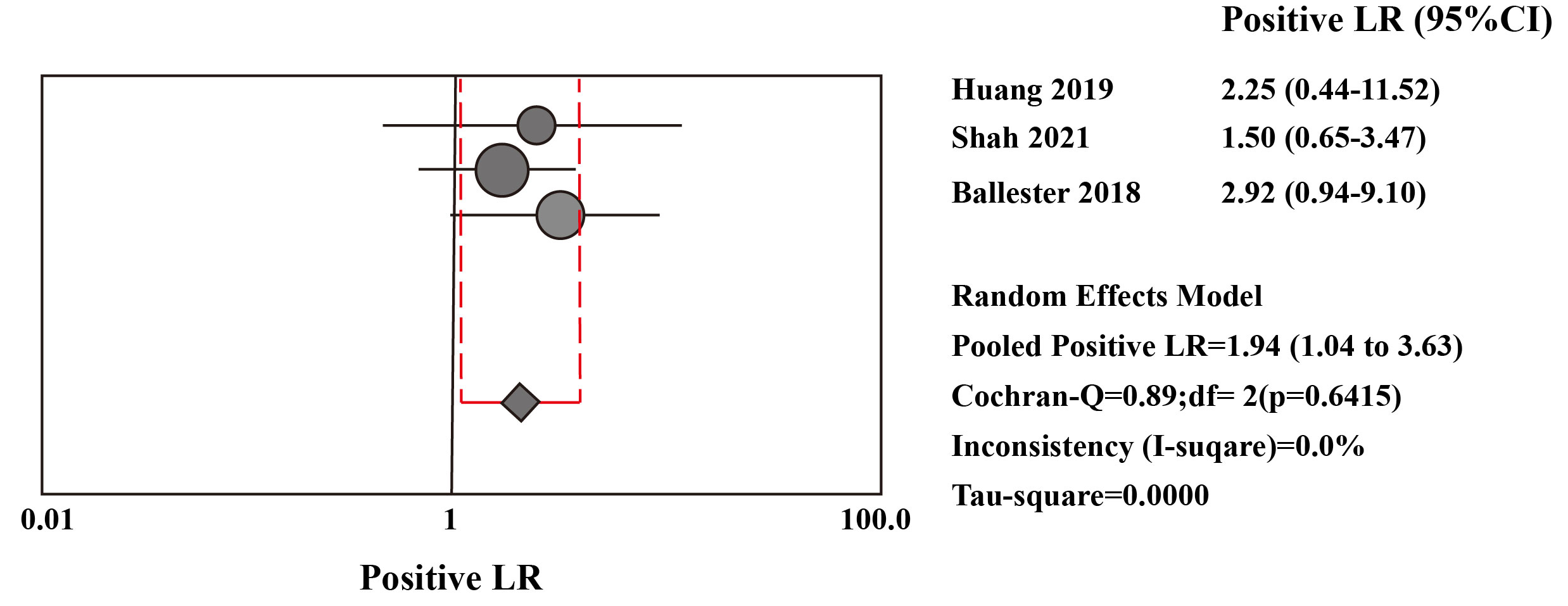

Supplement: Supplementary Figure 1 — The positive likelihood ratio of CSF liquid biopsy for LM diagnosis [file Image_1.jpeg]

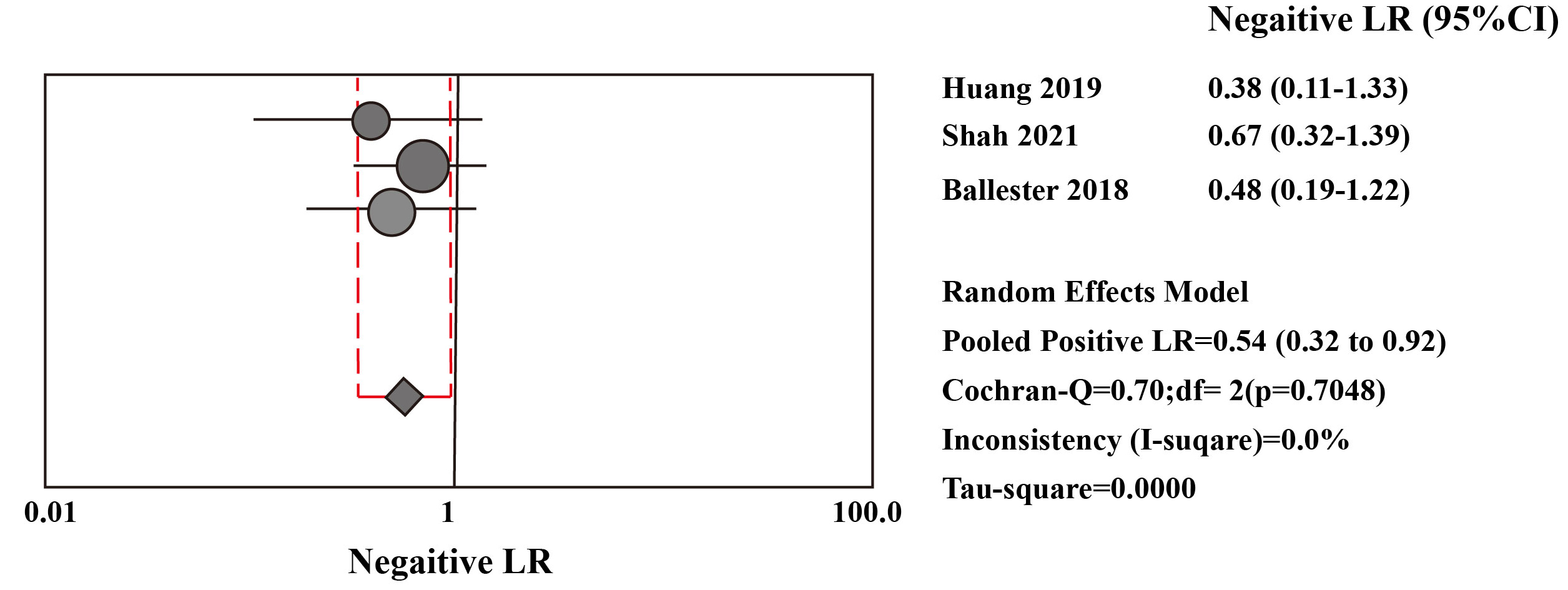

Supplement: Supplementary Figure 2 — The negative likelihood ratio of CSF liquid biopsy for diagnosis of LM. [file Image_2.jpeg]

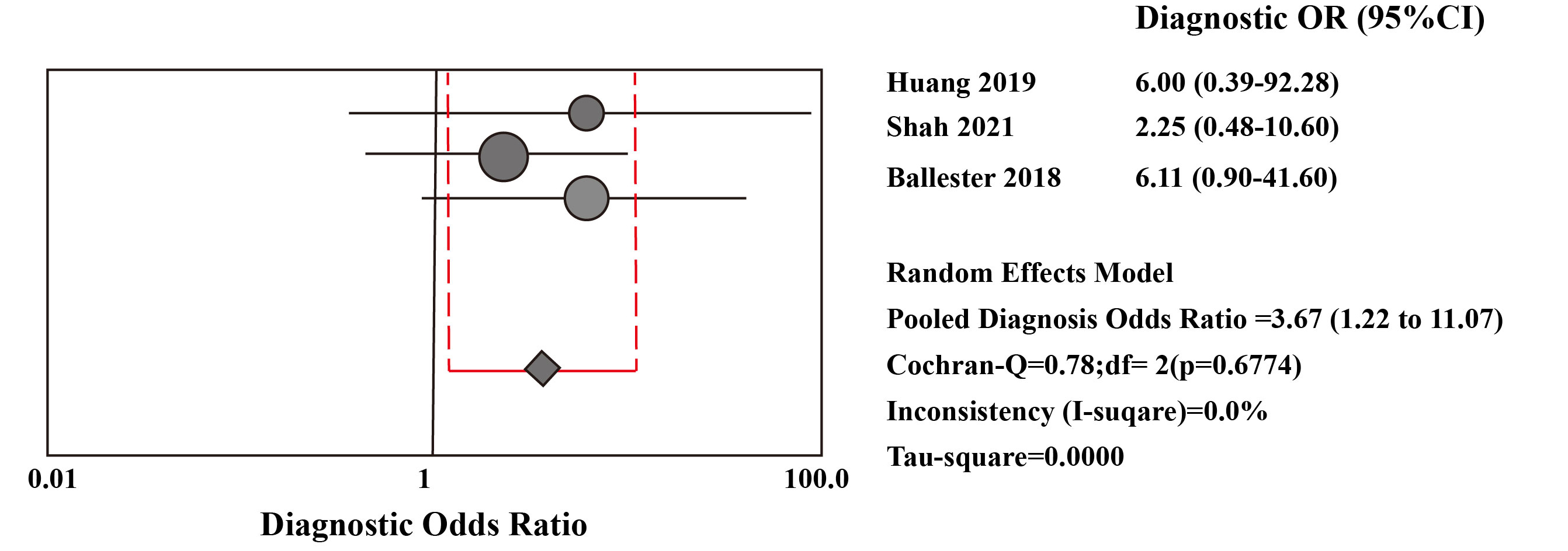

Supplement: Supplementary Figure 3 — Diagnostic odds ratios for the diagnosis of LM by CSF liquid biopsy. [file Image_3.jpeg]
